# Intimate Partner Violence during Pregnancy and Postpartum Depression in Japan: A Cross-sectional Study

**DOI:** 10.3389/fpubh.2017.00081

**Published:** 2017-04-24

**Authors:** Ayano Miura, Takeo Fujiwara

**Affiliations:** ^1^Department of Social Medicine, National Research Institute for Child Health and Development, Tokyo, Japan; ^2^Department of Global Health Promotion, Tokyo Medical and Dental University, Tokyo, Japan

**Keywords:** intimate partner violence, postpartum depression, the Edinburgh Postnatal Depression Scale, domestic violence, Japan

## Abstract

**Background:**

The impact of intimate partner violence (IPV) on postpartum depression (PPD) has been reported in various countries by many studies. However, the association between IPV and PPD in Japan has been scarce. In addition to the limited number of research on the relationship between IPV and PPD, the number of women seeking help from IPV support centers has been steadily increasing in Japan. Hence, it is of interest to explore the relationship between IPV during pregnancy and PPD in Japan.

**Methods:**

Four-page questionnaires assessing sociodemographic characteristics, women’s personal situation during pregnancy, and PPD were mailed to participants prior to the checkup and collected at the checkup sites or mailed back to the health center. Of 9,707 eligible mothers, 6,590 responded to a questionnaire at a 3- or 4-month infant health checkup (response rate: 68%). Verbal and physical IPV from partners was assessed with two questions in the questionnaire. Logistic regression analysis was conducted. PPD was evaluated using the Edinburgh Postnatal Depression Scale (EPDS) with a cutoff score of 8/9.

**Results:**

Partners’ verbal and physical abuse during pregnancy was significantly associated with PPD after adjusting for possible confounders. Specifically, odds ratios (ORs) of PPD for women who had been verbally abused by their partners during pregnancy at a frequency of “often” were 4.85 (95% CI, 2.23–10.55). ORs of PPD among women who had been physically abused by their partners during pregnancy at a frequency of “sometimes or often” were 7.05 (95% CI, 2.76–17.98). A positive dose-response relationship between both types of IPV and PPD was statistically significant (both *p* < 0.001). In addition, about 80% of physically abused women reported being verbally abused as well, indicating that these forms of IPV were highly comorbid.

**Conclusion:**

Both verbal and physical IPV during pregnancy is associated with PPD in Japan. This is the first study investigating the impact of IPV on PPD using a large number of subjects in the country. Further study using the same participants of the current study would allow us to explore the causality between IPV during pregnancy and PPD.

## Introduction

Postpartum depression (PPD) is a major depressive disorder that occurs during pregnancy or within 4 weeks of delivery (1) with a worldwide prevalence of approximately 10–15% (2). PPD is a serious obstetric complication that can increase the risk of suicide, and suicide accounts for 28% of maternal deaths that occur during pregnancy and 42 days after delivery (3–5). It is also well known that the condition has adverse effects on children that are attributable to poor parenting, such as child abuse and neglect (6), mothers’ impaired interactions with their infants [e.g., less vocal and visual communication, less smiling (7), being less sensitively attuned to their infants’ needs (8), or early discontinuation of breastfeeding (9)]. Other research has highlighted the long-term influence of PPD on children, such as an association between PPD and an increased risk of children’s emotional problems, as well as externalizing behaviors in later life (10). Thus, PPD should be considered as a detrimental perinatal health complication for childbearing women and those after childbirth that could negatively affect both mothers and children.

Investigating risk factors for the development of PPD is vital to its prevention. In fact, several risk factors have already been identified, such as being a single mother (11), low socioeconomic status (11), adverse life events (12), unwanted pregnancies (13), and intimate partner violence (IPV) during pregnancy (14–18). Of these risk factors, very little research on the association between IPV during pregnancy and PPD has been reported in Japan, even though a positive association between the two has been shown in other countries (15–19). Although the association between IPV and PDD was reported in several countries, as public health system factors can influence both IPV and PPD (20, 21), the degree of impact of IPV on PPD may differ. Furthermore, although a longitudinal study in Japan reported that IPV during pregnancy was associated with PPD (19), the sample size of this study was relatively small. Therefore, it is important to investigate the relationship between IPV and PPD using a larger sample size. Furthermore, a report from the Japanese government states that the number of women seeking help from IPV support centers has been steadily increasing (22), even though the 15% prevalence of IPV in Japan (23) is relatively low (20, 24). Thus, using a large number of subjects, we aimed to investigate the association between IPV during pregnancy and PPD in Japan.

## Patients and Methods

### Participants and Procedures

In Japan, municipal governments are responsible for promoting maternal and child health based on the Maternal and Child Health Act, including health checkups for children. All municipalities in Aichi, Japan, were recruited in this study. Of the 54 municipalities, 45 municipalities including Nagoya city, the prefectural capital of Aichi, agreed to participate in this study. The target sample were mothers (*N* = 9,707) who enrolled in a 3- or 4-month infant health checkup program conducted by municipal governments at public health centers from October to November 2012. The participation rate of women in the checkup program was 97.9%, and 6,590 women responded to the 4-page questionnaire, which collected information on demographics, the woman’s personal situation during pregnancy including IPV, PPD, and other social support (the overall response rate was 68%, with each center’s response rate ranging from 24.2 to 81.0%). The questionnaire was collected as follows: for 34 municipalities, the anonymous questionnaire was sent out to each participant *via* mail prior to the checkup, and the completed forms were collected at the checkup sites; for the remaining 11 municipalities, the questionnaire was distributed at the checkup sites and the participants mailed their completed forms back to the health center.

### Assessment of IPV during Pregnancy

Intimate partner violence during pregnancy was assessed based on two questions in the questionnaire: one question focused on verbal IPV and the other on physical IPV. The question on verbal IPV was: “Have you been verbally humiliated or yelled at by your partner during pregnancy?” The question on physical IPV was: “Have you been slapped or beaten up by your partner during pregnancy while having a fight?” Four response items were used for these questions: (1) “never,” (2) “a few times,” (3) “sometimes,” and (4) “often.” These questions on IPV were originally created specifically for this study, that is, they were not validated before the study. However, the questions were developed based on the revised Conflict Tactics Scale (25), and to minimize the burden on participants, only two questions were developed.

### Assessment of PPD

Postpartum depression was evaluated using the Edinburgh postnatal Depression Scale (EPDS), a 10-item questionnaire with a total score ranging from 0 to 30 (26). The EPDS is the most widely used self-report questionnaire to screen PPD (27), and various cutoff scores have been used in different studies (21, 28, 29). The current study used the cutoff score of 9 or more, based on one study which investigated the reliability and validity of the EPDS among women in the postnatal and control groups in Japan, and showed that sensitivity is 75% and specificity is 93% when using the cutoff score of 9 or more (30).

### Covariates

The following covariates were also assessed in the questionnaire: maternal age, marital status (i.e., married, unmarried, divorced, widowed, or others), whether expectant mothers had someone who supported them during pregnancy, maternal depressive symptoms (i.e., experiencing insomnia, being irritated, crying easily, or not being motivated to do anything for 2 weeks or longer) during one year prior to the diagnosis of pregnancy, maternal history of mental disorders (i.e., one question asked if women had previously been diagnosed with a mental illness or had received treatment for a mental illness before getting pregnant), subjective economic status (i.e., stable, able to manage, difficult to manage, or unstable), maternal and paternal employment status (i.e., working full-time, working part-time, or not working, which includes unemployed status, housewives, and students), sex of the child, whether the child is the mother’s first, low birth weight, preterm, maternal smoking history, maternal drinking history, and history of miscarriage and termination of pregnancy.

### Statistical Analysis

Of the women enrolled in the infant checkup program, those who responded to the EPDS were used for the analysis in this study (*N* = 6,534). Regarding the physical IPV variable, due to the small number of participants who answered “sometimes” and “often” (i.e., sometimes = 19, often = 6), these numbers were collapsed into the analysis.

Associations between IPV during pregnancy and PPD were assessed by multiple logistic regression analysis adjusted for covariates. Paternal age was omitted as a covariate because of a high correlation with maternal age (*r* = 0.68). In addition to the crude model, three adjusted models were employed. Model 1 was adjusted for covariates on demographic and social characteristics, that is, maternal age, marital status, whether expectant mothers had someone who supported them during pregnancy, maternal depressive symptoms during one year prior to the diagnosis of pregnancy, maternal history of mental disorders, subjective economic status, and employment status for mothers and fathers. Model 2 was further adjusted for covariates in model 1 plus perinatal covariates, that is, sex of the child, first child, low birth weight, preterm, maternal smoking history, maternal drinking history, and history of miscarriage and termination of pregnancy. Finally, model 3 was adjusted for covariates in model 2 plus both verbal and physical IPV simultaneously. A *p*-value <0.05 was considered to be statistically significant. All analyses were performed using the STATA/SE 13.1 software package (STATA Corporation, College Station, TX, USA).

## Results

### Demographic Data

Participants’ demographic data are shown in Table 1. Mean maternal age was 31.37 years, and around 88% of women were aged from 25 to 39. Mean paternal age was 33.32 years, and around 82% of men were aged from 25 to 39. Of the 6,534 women, 6,414 (98%) were married. Regarding subjective economic status, 734 (11%) considered their finances to be “difficult to manage” or “unstable.” When asked if women had experienced depressive symptoms during one year prior to the pregnancy, 955 (15%) confirmed that they did experience such symptoms. Regarding maternal support during pregnancy, 6,351 women (97%) indicated that they had someone to help them.

**Table 1:** **Characteristics of participants**.

		Total (*n* = 6,534)			
		*n* or mean	% or SD	Min.	Max.
Maternal age	Mean	31.37	4.8	16	47
	≤24	516	7.9		
	25–29	1,813	27.8		
	30–34	2,424	37.1		
	35–39	1,481	22.7		
	40≤	285	4.4		
	Missing	15	0.2		
Paternal age	Mean	33.32	5.6	16	60
	≤24	307	4.7		
	25–29	1,333	20.4		
	30–34	2,224	34.0		
	35–39	1,784	27.3		
	40≤	818	12.5		
	Missing	68	1.0		
Marital status	Married	6,414	98.2		
	Unmarried/divorced/widowed/others	102	1.6		
	Missing	18	0.3		
Verbal abuse from partner	None	5,793	88.7		
	A few times	436	6.7		
	Sometimes	243	3.7		
	Often	35	0.5		
	Missing	27	0.4		
Physical abuse from partner	None	6,444	98.6		
	A few times	53	0.8		
	Sometimes	19	0.3		
	Often	6	0.1		
	Missing	12	0.2		
EPDS score	≤8	5,911	90.5		
	9+	623	9.5		
Financial status	Stable	2,874	44.0		
	Able to manage	2,651	40.6		
	Difficult to manage or unstable	734	11.2		
	Missing	275	4.2		
Maternal employment status	Full-time	1,073	16.4		
	Part-time	314	4.8		
	Not working	5,055	77.4		
	Missing	92	1.4		
Paternal employment status	Full-time	6,294	96.3		
	Part-time	57	0.9		
	Not working	56	0.9		
	Missing	127	1.9		
Maternal depressive symptoms during one year prior to pregnancy	Yes	955	14.6		
	No	5,565	85.2		
	Missing	14	0.2		
Maternal history of mental disorders	Yes	98	1.5		
	No	413	6.3		
	Missing	6,023	92.2		
Maternal history of smoking	Yes	208	3.2		
	Stopped after pregnancy was confirmed	501	7.7		
	No	5,821	89.1		
	Missing	4	0.1		
Maternal history of drinking alcohol during pregnancy	Yes	257	3.9		
	No	6,266	95.9		
	Missing	11	0.2		
Having someone to provide support during pregnancy	Yes	6,351	97.2		
	No	162	2.5		
	Missing	21	0.3		
Pregnancy without any medical problems	Yes	5,329	81.6		
	No	1,157	17.7		
	Missing	48	0.7		
Sex of the child	Male	3,291	50.4		
	Female	3,183	48.7		
	Missing	60	0.9		
Pregnant with first child	Yes	3,228	49.4		
	No	3,306	50.6		
Birth weight of the child	<2,500	543	8.3		
	2,500 g≤	5,964	91.3		
	Missing	27	0.4		
Gestational week	<37 weeks	376	5.8		
	37 weeks≤	6,025	92.2		
	Missing	133	2.0		
History of miscarriage	0	5,375	82.3		
	1	929	14.2		
	2≤	230	3.5		
History of termination of pregnancy	0	6,082	93.1		
	1	349	5.3		
	2≤	103	1.6		

### IPV during Pregnancy and the EPDS Score

Regarding verbal IPV during pregnancy, 436 women (6.7%) reported frequency of verbal IPV as “a few times,” 243 (3.7%) reported “sometimes,” and 35 (0.5%) reported “often.” As to physical IPV during pregnancy, 53 women (0.8%) answered with “a few times,” and 25 (0.4%) answered “sometimes” or “often.” The distribution of the EPDS score showed that 623 women (9.5%) scored 9 and above, and therefore, this group could be considered to have PPD.

### The Association between IPV during Pregnancy and PPD

The association between verbal and physical IPV is shown in Table 2. Among those women who were physically abused a few times during pregnancy, 79.3% also reported being verbally abused at least a few times. Of those who were physically abused sometimes or often, 84.0% reported to be verbally abused at least a few times. These results suggest that both forms of IPV were highly comorbid.

**Table 2:** **Association between verbal intimate partner violence (IPV) and physical IPV during pregnancy**.

		Verbal IPV					
		Never	A few times	Sometimes	Often	No response	Total
Physical IPV	Never (%)	5,777 (89.7)	416 (6.5)	208 (3.2)	25 (0.4)	18 (0.3)	6,444
	A few times (%)	10 (18.9)	17 (32.1)	21 (39.6)	4 (7.6)	1 (1.9)	53
	Sometimes/often (%)	4 (16.0)	3 (12.0)	13 (52.0)	5 (20.0)	0 (0.0)	25
	No response (%)	2 (16.7)	0 (0.0)	1 (8.3)	1 (8.3)	8 (66.7)	12

	Total	5,793	436	243	35	27	6,534

Table 3 shows the association between IPV during pregnancy and PPD. The crude model showed that partners’ verbal and physical abuse was significantly associated with PPD (both *p* < 0.001). Adjusting for covariates on demographic and social characteristics in model 1 produced significant results with the same trend remaining for both types of IPV. In model 2, which was further adjusted for perinatal covariates, significant results with the same trend were confirmed [verbal IPV: odds ratios (ORs) for “a few times”: 2.25 (95% CI, 1.70–2.96), OR for “sometimes”: 2.79 (95% CI, 2.00–3.89), and OR for “often”: 4.85 (95% CI, 2.23–10.55); physical IPV: OR for “a few times”: 2.74 (95% CI, 1.42–5.25), and OR for “sometimes or often”: 7.05 (95% CI, 2.76–17.98)]. All results for the *p* for trend showed <0.001. Model 3 was adjusted for the same covariates used in model 2, plus both verbal and physical IPV. The results remained significant with the same trend, although ORs were attenuated for physical IPV.

**Table 3:** **Association between intimate partner violence (IPV) during pregnancy and postpartum depression**.

		Crude model (*n* = 6,534)			Model 1 (*n* = 6,534)			Model 2 (*n* = 6,529)			Model 3 (*n* = 6,529)		
		Odds ratio (OR)	95% CI	*p*-Value	Adjusted OR	95% CI	*p*-Value	Adjusted OR	95% CI	*p*-Value	Adjusted OR	95% CI	*p*-Value
Verbal abuse from partner	Never	Reference			Reference			Reference			Reference		
	A few times	3.18	2.47–4.09	<0.001	2.2	1.67–2.89	<0.001	2.25	1.70–2.96	<0.001	2.19	1.65–2.90	<0.001
	Sometimes	5.35	4.01–7.15	<0.001	2.86	2.06–3.97	<0.001	2.79	2.00–3.89	<0.001	2.49	1.76–3.53	<0.001
	Often	11.55	5.91–22.56	<0.001	4.82	2.28–10.20	<0.001	4.85	2.23–10.55	<0.001	4.04	1.81–9.03	0.001
	No response	2.13	0.73–6.18	0.17	1.00	0.29–3.43	>0.99	0.95	0.28–3.28	0.94	1.11	0.28–4.46	0.88
	*p* for trend	<0.001			<0.001			<0.001			<0.001		
Physical abuse from partner	Never	Reference			Reference			Reference			Reference		
	A few times	5.13	2.89–9.12	<0.001	2.67	1.40–5.09	0.003	2.74	1.42–5.25	0.002	1.51	0.77–2.99	0.23
	Sometimes/often	14.97	6.69–33.46	<0.001	8.11	3.22–20.41	<0.001	7.05	2.76–17.98	<0.001	3.85	1.48–10.02	0.006
	No response	3.33	0.90–12.32	0.072	0.94	0.20–4.46	0.94	0.82	0.17–3.94	0.808	0.69	0.11–4.40	0.7
	*p* for trend	<0.001			<0.001			<0.001			0.005		

*Model 1 was adjusted for maternal age, marital status, whether expectant mothers had someone who supported them during pregnancy, maternal depressive symptoms during the one year prior to diagnosis of pregnancy, maternal history of mental disorders, subjective economic status, and maternal and paternal employment status*.

*Model 2 was adjusted for covariates in model 1 plus sex of the child, whether the child was the mother’s first child, low birth weight, preterm, maternal smoking history, maternal drinking history, and history of miscarriage and termination of pregnancy*.

*Model 3 was adjusted for covariates in model 2 plus both verbal and physical IPV*.

## Discussion

To the best of our knowledge, this is the first study investigating the relationship between IPV during pregnancy and PPD using a large number of subjects in Japan. Consistent with previous research conducted in Japan (19) and studies from other countries (15–18, 31), the current study showed the association between verbal or physical IPV and PPD. These tendencies remained after being adjusted for possible covariates.

The present study has several limitations. First, this study is cross sectional, which means that recall bias for IPV during pregnancy is unavoidable. Thus, it is not possible to determine the causality between IPV and PPD. It is possible that antenatal IPV contributed to the development of PPD, or women with a higher risk of developing PPD tend to have abusive partners. Second, we did not assess the EPDS score during pregnancy. Because of this, we could not exclude women who simply had PPD and thus scored 9 and above in the EPDS at 3 or 4 months postpartum. This may have caused an overestimation of the impact of IPV during pregnancy on PPD. Third, even though the EPDS is the most widely used screening instrument (27), it is not a diagnostic tool for PPD. Hence, the criteria for PPD used in this study would be less precise than the clinically used criteria, such as the Diagnostic and Statistical Manual of Mental Health Disorders (DSM-V). Fourth, unlike other research that used validated questions in measuring IPV [e.g., the Demographic and Health Surveys (DHS), the International Violence against Women Surveys (IVAWS), or a standardized structured questionnaire that was piloted in all countries having been surveyed] (24, 32), the current study used original questions to assess IPV during pregnancy. This makes it harder to directly compare our results with previous research on the same dimensions. Fifth, other possible risk factors for both IPV and PPD, such as disability (33), were not assessed in this study, thus current findings may not be causal.

Despite these limitations, the current study offers some insights into the significant influence of both verbal and physical IPV during pregnancy on PPD. Regular screening for antenatal IPV by public health nurses could identify those women who need further support, such as referral to women’s guidance centers for confidential advice and support. In Japan, most pregnant mothers are supposed to register their pregnancy at a public health center, and public health nurses can later visit their home if needed. Thus, through this system, it is possible to screen IPV status during pregnancy during a visit by a public health nurse, who can then provide a referral to support services. An evaluation of the effectiveness of this intervention to reduce PPD prevalence is needed. Further, one direction for future research would be to conduct another longitudinal study to infer the causal relationship between IPV and PDD. Assessing expectant mothers with the EPDS might improve the evaluation of the impact of IPV during pregnancy on PPD. In addition, it might be possible to determine the precise number of women with PPD by using both the EPDS and DSM-V. Using validated questionnaires such as the DHS, IVAWS, or the one used in WHO study (24) would help to directly compare the current findings with previous research.

Our findings have some significant clinical implications on PPD and its prevention. As the experience of verbal or physical IPV during pregnancy may increase the likelihood of PPD onset, healthcare providers should screen women for IPV during pregnancy to identify those at a higher risk of developing PPD due to IPV, such as during home visits by a public health nurse. As previous research has shown that treating depressed mothers during pregnancy can prevent depression after childbirth (34), PPD screening during pregnancy should be acknowledged as a feasible and promising preventative intervention. That is, establishing PPD-screening programs and offering proper support for women in need could help improve women’s perinatal mental health and emotional wellbeing.

That physical IPV during pregnancy had a stronger impact on developing PPD than did verbal IPV gives us some insights into the relationship between IPV and PPD. Physical IPV during pregnancy seems to have a more devastating outcome than verbal IPV, as verbal abuse during pregnancy does not leave any visible trauma on pregnant women and their unborn children directly. However, a study in the United States asserted that psychological abuse has a negative emotional influence on relationships that is as damaging as physical abuse (35). In addition, it has been revealed that psychological abuse almost always precedes physical abuse (36). Similarly, our study found that verbal and physical IPV were highly comorbid. Together with findings from previous research, our study highlights the importance of early detection of IPV, including verbal IPV, which is likely to occur before physical IPV, in order to minimize or prevent negative consequences such as PPD.

## Ethics Statement

This study was approved by the Ethics Committee of the National Center for Child Health and Development (reference number: 611). The Ethics Committee approved that personal informed consent could be exempted if participants responded anonymously to the questionnaire.

## Author Contributions

TF conceived the study and collected the data; finalized the manuscript. AM analyzed and drafted the first draft. Both authors approved the final manuscript.

## Conflict of Interest Statement

The authors declare that the research was conducted in the absence of any commercial or financial relationships that could be construed as a potential conflict of interest.
